# Non-Small-Cell Lung Cancers (NSCLCs) Harboring RET Gene Fusion, from Their Discovery to the Advent of New Selective Potent RET Inhibitors: “Shadows and Fogs”

**DOI:** 10.3390/cancers16162877

**Published:** 2024-08-19

**Authors:** Gianluca Spitaleri, Pamela Trillo Aliaga, Ilaria Attili, Ester Del Signore, Carla Corvaja, Gloria Pellizzari, Jalissa Katrini, Antonio Passaro, Filippo de Marinis

**Affiliations:** 1 Division of Thoracic Oncology, European Institute of Oncology (IEO), IRCCS, Via Ripamonti 435, 20141 Milan, Italy; 2 Division of New Drugs and Early Drug Development for Innovative Therapies, European Institute of Oncology, IRCCS, 20141 Milan, Italy; 3 Department of Oncology and Haematology (DIPO), University of Milan, 20122 Milan, Italy

**Keywords:** NSCLC, RET, RET gene fusion, RET multi-targeted inhibitors, new selective RET inhibitors, pralsetinib, selpercatinib, toxicity, resistance mechanism, new drugs

## Abstract

**Simple Summary:**

RET fusions are relatively rare in Non-Small-Cell Lung Cancers (NSCLCs), being around 1–2% of all NSCLCs. Chemotherapy and immunotherapy have a low impact on the prognosis of patients with RET fusions positive NSCLC. Multitargeted RET inhibitors have shown modest activity jeopardized by high toxicity. New potent and selective RET inhibitors (RET-Is) (pralsetinib and selpercatinib) have achieved a higher efficacy in minimizing the known toxicities of the old drugs. This review will focus on the advent of new potent and selective RET-Is. We will describe their efficacy as well as the main mechanisms of resistance to them. We will further proceed to deal with new drugs and strategies proposed to overcome the resistance to RET-Is. In the last section, we will also focus on the safety profile of RET-Is, dealing with the main toxicities as well as the rare but severe adverse events.

**Abstract:**

RET fusions are relatively rare in Non-Small-Cell Lung Cancers (NSCLCs), being around 1–2% of all NSCLCs. They share the same clinical features as the other fusion-driven NSCLC patients, as follows: younger age, adenocarcinoma histology, low exposure to tobacco, and high risk of spreading to the brain. Chemotherapy and immunotherapy have a low impact on the prognosis of these patients. Multitargeted RET inhibitors have shown modest activity jeopardized by high toxicity. New potent and selective RET inhibitors (RET-Is) (pralsetinib and selpercatinib) have achieved a higher efficacy minimizing the known toxicities of the multitargeted agents. This review will describe the sensitivity of immune-checkpoint inhibitors (ICIs) in RET fusion + NSCLC patients, as well their experiences with the ‘old’ multi-targeted RET inhibitors. This review will focus on the advent of new potent and selective RET-Is. We will describe their efficacy as well as the main mechanisms of resistance to them. We will further proceed to deal with the new drugs and strategies proposed to overcome the resistance to RET-Is. In the last section, we will also focus on the safety profile of RET-Is, dealing with the main toxicities as well as the rare but severe adverse events.

## 1. Introduction

**Figure 1:**
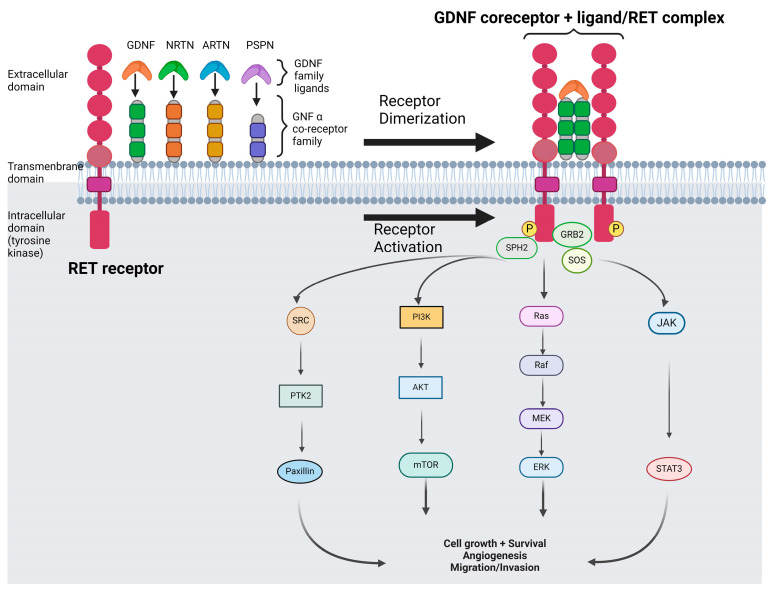
Representation of RET and the GDNF (glial cell line-derived neurotrophic factor) co-receptor-a family with their respective ligands. The interaction of the ligands with the co-receptors leads to homodimerization with RET and its subsequent activation ((‘GDNF coreceptor + ligand/RET homodimer’ complex). RET activation leads to the activation of several intracellular pathways. Abbreviations: RET = rearranged during transfection protooncogene; GDNF = glial cell line-derived neurotrophic factor; NRTN = neurturin,; ARTN = artemin, PSPN = persephin; P = phosphorylated; GRB2: growth-factor-receptor-bound protein 2; SHP2 = SRC homology 2; SOS = son-of-sevenless protein; SRC = a non-receptor tyrosine kinase protein SRC short for sarcoma; PTK2 = protein tyrosine kinase 2; PI3K = phosphatidylinositol 3-kinase; AKT = protein kinase B; mTOR = mammalian target of rapamycin; RAS = rat sarcoma gene; RAF = rapidly accelerated fibrosarcoma gene; MEK = mitogen-activated protein kinase; ERK = extracellular signal-regulated kinase; JAK = Janus kinase protein; STAT3 = signal transducer and activator of transcription 3 protein. Created with BioRender.com (accessed on 14 July 2024).

RET (rearranged during transfection) protooncogene was discovered during the transfection of mouse NIH3T3 cells with human lymphoma DNA in 1985 [1]. The RET protooncogene is located on Chromosome 10q11.2 and encodes a transmembrane tyrosine kinase (TK) that consists of a large extracellular domain, a transmembrane domain, and an intracellular tyrosine kinase domain. Among the two isoforms derived from alternative splicing (RET9 and RET51), RET51 seems to be the more common isoform in cancers [2,3,4]. RET belongs to the glial cell line-derived neurotrophic factor (GDNF) coreceptor-a family; these co-receptors bind several ligands named as GDNFs (neurturin, artemin, and per-sephin), and after interaction with these ligands, they activate RET through its homodimerization (‘GDNF coreceptor + ligand/RET homodimer’ complex) and its subsequent activation of the RET tyrosine kinase domain [5,6]. The phosphorylation of several RET sites can activate the following different corresponding intracellular pathways: Y1062 (Ras/MAPK, PI3K/AKT, and JNK), Y1096 (Ras/MAPK and PI3K/AKT), Y1015 (protein kinase C), Y752 and Y928 (STAT3), and Y687, and Y981 (SHP2 and SRC kinase) [7,8,9,10,11,12,13] (Figure 1).

RET protooncogene is physiologically expressed during the fetal life and it is essential for the development of the intestine, the kidney, and the nervous system (mainly neural crest cells and motoneurons), as well for the regulation and function of hematopoietic cells and spermatogenesis. In humans, many syndromes are related to RET mutations, as follows: Hirschsprung disease, congenital anomalies of the kidney or urinary tract, and congenital central hypoventilation syndrome [14,15,16,17,18,19,20].

RET can be expressed and activated in several cancers through point mutations or rearrangements, causing fusion genes. These alterations cause RET activation in tumor cells, even in the absence of ligand-coreceptor GDFN interaction. To cause a functional RET fusion, the breakpoints must occur within intron 11 and must lead to fusions with only the cytoplasmic portion of RET containing the tyrosine kinase domain. At least 35 genes have been reported to be a partner gene of the RET fusions. RET fusions were initially discovered in papillary thyroid carcinoma in 1987, and their incidence is around 10–20% in this disease [21]. In non-small-cell lung carcinoma (NSCLC), they were discovered in 2012 [22,23]. In NSCLC, their incidence is relatively rare, being around 1–2% of all NSCLCs, with an estimated 10,000 new cases each year around the world. The most common partner genes of RET fusions in NSCLC are KIF5B (which encodes the kinesin family member 5B) (83.6%) and CCDC6 (15.1%) [21,24,25].

According to the ESMO (European Society Medical Oncology) guidelines, in those tumors where RET fusions or mutations are relatively common, FISH (Fluorescence in situ hybridization) or RT-PCR (Reverse-Transcription-Polymerase Chain Reaction) could be used. In cancers where RET fusions are rare, such as NSCLC, next-generation sequencing (NGS) with broad panel assays are recommended to allow for screening in a histotype-agnostic manner [26]. Immunohistochemistry (IHC) is not reliable for the detection of RET rearrangement due to relatively high percentages of false negatives (around 46%) and false positives (around 62%) in specimens previously tested using RT-PCR. RT-PCR and FISH together are sensitive and effective tools. However, RT-PCR is insufficient to detect novel fusion partners. In reverse, NGS (especially those with hybrid DNA/RNA-based platforms) has the advantage to detect novel fusions, reveal the gene partner, and to be adequate to find both the gene fusions and/or the somatic mutations [27].

The clinical features of patients with NSCLC harboring RET fusion (RET fusion + NSCLC) include younger age (median age around 60 years), equal prevalence in both sexes, adenocarcinoma histology, poorly differentiated tumors, and no or low exposure to tobacco [28,29]. Moreover, concerning the spreading to the central nervous system (CNS), RET disease shows an intermediate behavior between ALK (anaplastic lymphoma kinase) + and ROS1(ROS proto-oncogene 1) + NSCLC patients; the incidence of brain metastases at the diagnosis was 25% in a retrospective analysis of 185 patients with RET fusion + NSCLC, and around 46% of them developed CNS metastases during their lifespan [30]. It is not known what the exact mechanisms are which could explain the brain tropism of RET fusion-positive NSCLC cancers. However, in NSCLC cancers spreading to the brain, several mechanisms have been observed that are potentially involved with organ-tropism, such as the epithelial–mesenchymal transition mechanism (loss of E-cadherin, overexpression of vimentin and N-cadherin), the activation of migration-related chemokines (high CXCL12 and its receptor CXCR4 levels), and the activation of EGFR/MET/VEGF pathways [31].

The data from retrospective studies have shown that chemotherapy has modest activity, but patients with RET fusion + NSCLC are likely to be sensitive to pemetrexed-based regimens (Table S1) [32,33,34,35].

This review describes the limited activity of the immune-checkpoint inhibitors (ICIs) in RET fusion + NSCLC patients, as well their experiences with the ‘old’ multi-targeted RET inhibitors. This review will focus on the advent of new potent and selective RET-Is. We will describe their efficacy as well as the main mechanisms of resistance to them. We will further proceed to deal with the new drugs and strategies proposed to overcome the resistance to RET-Is. In the last section, we will also focus on the safety profile of RET-Is, dealing with the main toxicities as well as the rare but severe adverse events.

## 2. Immunotherapy

The treatment landscape and prognosis of NSCLC patients has been revolutionized with the advent of ICIs. However, the benefit of this treatment in patients with gene-addicted NSCLC like RET fusion + NSCLC is controversial [36]. The data regarding immunotherapy in patients with RET fusion + NSCLC come not from prospective, but from retrospective studies.

In a large retrospective study of patients with solid tumors harboring RET aberrations, 70 received systemic therapy for advanced disease: 20 (29%) received ICI and 50 (71%) received non-ICI-based regimens. Overall, non-ICI regimens were associated with decreased risk for treatment discontinuation compared with ICI (HR = 0.31; 95% CI 0.16–0.62; *p* = 0.000834). Moreover, a statistical trend was observed for detrimental outcome when patients with RET fusion-driven solid tumors were treated with ICI compared to other regimens. Among the 18 evaluable patients (15 with NSCLC) for Programmed Cell Death Ligand 1 (PD-L1) expression, around 80% of them had tumors with negative or intermediate expression of PD-L1. Two patients with PD-L1 overexpressed NSCLC had to discontinue pembrolizumab due to disease progression within 2 months. Moreover, all evaluable patients (15 patients) had a low median tumor mutational burden (mTMB) (≤5/megabase, Mb) and microsatellite-stable tumors (10 evaluable patients) [37].

Focusing on NSCLC, in a distinct retrospective monocentric study of 74 patients with RET fusion + NSCLC, the authors reported that most of the evaluable patients (81%, 21/26) were PD-L1 negative (58%, 15/26) or intermediate (<50%) (23%, 6/26), and among the 44 patients with sufficient tumor sample for TMB, the mTMB was significantly lower in respect to that of 3631 RET wild-type NSCLC patients (1.75 vs. 5.27 mutations/Mb) [38]. In total, 13 out of 16 patients treated with ICI (mostly as a second-line setting) were evaluated for efficacy, but no responses were recorded, the median progression-free survival (mPFS) was 3.4 months, and neither PD-L1 overexpression nor high mTMB were associated with a better clinical outcome [38].

In a recent Chinese retrospective study of 232 patients with RET fusion + NSCLC, they confirmed that most of them (>80%) were PD-L1 negative or intermediate, and, among the 35 evaluable patients, 86% had low m-TMB (<10 mutations per Mb). Moreover, 38 of them were treated with ICI (17 in the first-line setting and 21 in the second-line setting), and the overall response rate (ORR) was 26.3%, the mPFS was 5 months, and the median overall survival (OS) was 19 months. Therefore, this study confirmed that PD-L1 overexpression did not augment the ORR with ICI [39]. We can speculate that the high expression of PD-L1 did not influence the response to immunotherapy because tumors harboring RET gene fusion are defined as cold tumors since they are associated with low TMB, genomic stability, low numbers of tumor-infiltrating lymphocytes, and low numbers of tertiary lymphoid structures [40].

Three retrospective analyses of ICIs among patients with NSCLCs harboring molecular gene drivers (including as well RET fusion genes) confirmed the poor outcome of patients with RET fusion + NSCLCs when treated with this type of treatment. In the IMMUNOTARGET registry, in 16 patients with RET fusion + NSCLC treated with ICIs (second-line or further setting), the ORR was 6% with a mPFS of 2.1 months [41]. In a retrospective analysis of ICIs among patients with NSCLCs harboring rare targetable drivers, 4 out of 13 patients with RET fusion + NSCLCs were treated with ICIs with no responses, and an mPFS of 3 months was recorded [42]. The GFPC 01-2018 study enrolled nine patients with RET fusion + NSCLCs, recording an ORR of 37.5% and an mPFS of 7.6 months. Of note, five patients (55%) were former smokers [43]. Table 1 summarizes the results of multiple retrospective studies of patients with RET fusion + NSCLCs who were treated with ICIs.

## 3. Multitargeted Agents

Phase II trials of various multi-tyrosine kinase inhibitors (TKIs) in patients with RET fusion + NSCLCs have demonstrated that these drugs have modest activity accompanied by high toxicity, very likely associated with non-RET targets’ inhibition (especially VEGFR-2 inhibition). Table 2 depicts the main results of these phase II trials of multi-TKIs.

### 3.1. Cabozantinib

Cabozantinib is a multi-TKI with activity towards MET (Mesenchymal–Epithelial Transition), AXL, VEGFR2 (Vascular Endothelial Growth Factor Receptor-2), FLT3 (FMS-like tyrosine kinase 3), and c-KIT (or stem cell factor receptor) in addition to RET. It has been approved in different diseases such as renal cell carcinoma, medullary thyroid cancer, and hepatocellular carcinoma [44]. In a small phase II trial, 26 patients with RET fusion + (FISH or NGS detected) NSCLCs were treated with cabozantinib (60 mg daily), attaining an ORR (principal endpoint of the trial) of 28% (7/25), a median duration of response (mDoR) of 7 months, a mPFS of 5.5 months, and an OS of 9.9 months. Interestingly, the efficacy of cabozantinib was negatively affected by the prior treatment’s line numbers, prior VEGF inhibitors, and gene partners (no responses were recorded in tumors harboring CCDC6-RET or ERC1-RET). The most common treatment-related adverse events (AEs) of any grade were increased alanine aminotransferase (ALT) (96%), increased aspartate aminotransferase (AST) (73%), hypothyroidism (69%), diarrhea (62%), palmar plantar erythrodysesthesia (58%), and skin hypopigmentation (50%). The G3/4 AE rate was 71%, and no treatment-related deaths were observed. The most common G ≥ 3 AEs were lipase elevation (four patients, 15%), increased ALT (two patients, 8%), AST (two patients, 8%), thrombocytopenia (two patients, 8%), and hypophosphatemia (two patients, 8%). AEs leading to dose reduction were 73%, and those leading to drug discontinuation were 8% [45].

### 3.2. Vandetanib

Vandetanib is an oral multi-TKI that inhibits RET, EGFR (Epidermal Growth Factor Receptor), and VEGFR-2/3, and is approved for the treatment of medullary thyroid carcinoma [46]. In the Japanese phase II trial LURET, 1536 patients with EGFR mutation-negative NSCLC were screened, of whom 34 were RET fusion + (2%) and 19 were previously treated patients (10 KIF5B-RET and 6 CCDC6-RET) who were enrolled and treated with vandetanib (300 mg daily), observing an ORR of 53% (9/17), a mDoR of 5.6 months, and a mPFS of 4.7 months. In an unlikely outcome for cabozantinib, patients with CCDC6-RET fusions were associated with longer mPFS compared to patients with KIF5B-RET fusions (8.3 vs. 2.9 months) [47]. At a median follow-up of 37.9 months, the OS was 13.5 months. The most common AEs were hypertension (84.2%), diarrhea (78.9%), and rash acneiform (63.2%). The G3/4 AE rate was 84% and no treatment-related deaths were recorded. The most common G3-4 AEs were hypertension (thirteen patients, 68.4%), diarrhea (two patients, 10.5%), skin rash (three patients, 15.8%), and QTc prolongation (two patients, 10.5%). AEs leading to dose reduction were 57.9%, those leading to dose interruption were 89.5%, and those leading to drug discontinuation were 21% [48].

In a small Korean phase II study, 18 previously pretreated patients with RET fusion + NSCLCs were treated with vandetanib (300 mg daily), attaining an ORR of 18% (3/17), an unreported mDoR, an mPFS of 4.5 months, and an OS of 11.6 months. No responses were observed in patients with KIF5B-RET fusions, and conflicting results were observed in two patients with CCD6C (one with partial response [PR] and one with mixed response). The most common AEs were hypertension (16 patients; 89%), skin rash (13 patients, 72%), diarrhea (8 patients, 44%), and acne (5 patients, 28%). The G3 toxicity rate was 34%. The G3 AEs were hypertension (three patients, 17%), QTc prolongation (two patients, 11%), and AST or ALT increase (one patient, 6%). There were no adverse events associated with grade 4 or 5 toxicity. AEs leading to dose reduction were 34%, those leading to dose interruption were 28%, and those leading to drug discontinuation were not reported [49].

### 3.3. Lenvatinib

Lenvatinib is a multi-TKI of VEGFR1–3, fibroblast growth factor receptor (FGFR1–4), platelet-derived growth factor receptor alpha (PDGFRα), RET, and KIT. Lenvatinib is approved for refractory-differentiated thyroid cancer, hepatocellular carcinoma, and renal cell carcinoma (in combination with everolimus) [50]. In a small Japanese phase II trial, 25 RET fusion + NSCLC (13 KIF5B-RET, 12 CCDC6-RET) patients were treated with lenvatinib (24 mg daily). The ORR was 16%, the mDoR was not estimable, the mPFS was 7.3 months, and the OS was not estimable at an unreported median follow-up. The responses were similar between KIF5B-RET and CCDC6-RET fusions; however, a higher disease control rate and a longer mPFS were associated with CCDC6-RET fusions. The most common AEs were hypertension (68%), nausea (60%), decreased appetite (52%), diarrhea (52%), proteinuria (48%), vomiting (44%), and headache (40%). The grade ≥ 3 AE rate was 92% (23/25). The most common grade ≥ 3 AEs were hypertension (fourteen patients, 56%), hyponatremia (five patients, 20%), proteinuria (four patients,16%), pneumonia (four patients, 16%), and nausea (three patients, 12%). One treatment-related death (pneumonitis) was reported. AEs leading to dose interruption were 76%, those leading to dose reduction were 64%, and those leading to drug discontinuation were 24% [51].

### 3.4. Ponatinib

Ponatinib is a multi-TKI that inhibits BCR-ABL (Breakpoint Cluster Region-Abelson murine Leukemia viral oncogene homolog 1) and RET and is approved for the treatment of chronic myeloid leukemia [52]. A phase II trial of ponatinib (30 mg daily) in previously treated patients with RET fusion + NSCLCs has been prematurely closed due to a slow accrual and lack of efficacy after enrolling the first nine patients. There were no responses (ORR 0%), the mPFS was 3.8 months, and the OS was 17.47 months. The most common AEs were skin rash (five patients, 56%), constipation (five patients, 56%), diarrhea (four patients, 44%), abdominal pain (two patients, 22%), nausea (two patients, 22%), and dry skin (two patients, 22%). The G3 AE rate was 11% (1 pt dry skin). AEs leading to dose interruption were 56%, those leading to dose reduction were 22%, and those leading to drug discontinuation were not reported [53].

**Table 2:** Phase II trials of multi-tyrosine kinase inhibitors in patients with RET fusion NSCLC.

Reference	Drug	Targets	Pts	Line	ORR	mPFS (Months)	OS (Months)	AE G3/4	Red.	Disc.
Drilon 2016 [45]	Cabozantinib	RET, MET, AXL, FLT3, c-KIT	26	≥1 L^%^	28%	5.5	9.9	71%	73%	8%
Yoh 2017/2021 [47,48]	Vandetanib	RET, EGFR, VEGFR2/3	19	≥2 L	53%	4.7	13.5	84%	58%	21%
Lee 2017 [49]	Vandetanib	RET, EGFR, VEGFR2/3	18	≥2 L	18%	4.5	11.6	34%	28%	NR
Hida 2019 [51]	Lenvatinib	RET, VEGFR1-3, FGFR1-4, PDGFRa, cKIT	25	≥1 L *	16%	7.3	NE	92%	64%	24%
Gainor 2020 [53]	Ponatinib	BCR-ABL-RET	9	≥2 L	0	3.8	17.5	11%	56%	NR

Legend: % 6 patients in 1st line; * only 2 patients in 1st line. Abbreviations: Pts = patients; ORR = overall response rate; mPFS = median progression-free survival; OS = median overall survival; AE: adverse events; G = grade; red. = reduction; disc. = discontinuation; RET = rearranged during transfection; MET (Mesenchymal–Epithelial Transition), VEGFR1-3 (Vascular Endothelial Growth Factor Receptor-1-3), FLT3 (FMS-like tyrosine kinase 3), c-KIT (or stem cell factor receptor); EGFR = Epidermal Growth Factor Receptor; FGFR1-4 = Fibroblast Growth Factor Receptor, PDGFR-α =platelet-derived growth factor receptor alpha; NR = not reported; NE = not evaluable.

### 3.5. Other Multi-TKIs

Poor data are available for the remaining multitargeted agents (sorafenib, sunitinib, alectinib, regorafenib, and nindetanib), and their activity seems to be very modest. Table S2 summarizes the clinical data of these multi-TKIs available from clinical trials to retrospective studies [33,54,55,56,57,58,59].

## 4. New and Selective RET Inhibitors (RET-Is)

### 4.1. Pralsetinib

Praseltinib (formerly known as BLU-667) in an oral selective and potent TKI of RET. In vitro, pralsetinib has been shown to inhibit RET (half-maximal inhibitory concentration, IC50 0.4 nmol/L) with a ≥10-fold increased potency when compared to other multi-TKIs (IC50 for cabozantinib was 11 nmol/L and vandetanib was 4 nmol/L) without targeting VEGFR2 (IC50 34 nmol/L) [60]. Moreover, pralsetinib demonstrated the same robust activity towards the CCDC6-RET fusion (IC50 0.4 nmol/L) and towards the acquired RET gatekeeper mutations (V804R/L), which could confer resistance to the multi-TKIs [61]. In preclinical models, pralsetinib has shown activity both against KIF5B–RET Ba/F3 and KIF5B–RETV804L Ba/F3 allograft tumors, and in the same work, the first proof of clinical activity towards 4 patients enrolled in the phase I/II trial ARROW was presented (two patients with medullary thyroid carcinoma and two patients with NSCLC, the first TKI-naïve and the second previously pretreated with vandetanib plus everolimus) [60].

ARROW was a global phase I/II trial of pralsetinib in patients with medullary thyroid cancer, RET-altered NSCLCs, and other RET-altered solid tumors [62]. During the Phase I Bayesian dose escalation, two dose-limiting toxicities (hypertension and hyponatremia) at 600 mg once daily had occurred, and the recommended dose for the phase II part was established as 400 mg once daily, as in the safety and pharmacokinetics data. The principal endpoints in phase II of the trial were ORR as assessed using blinded central review (BIRC) and safety. RET was tested locally. In total, 587 patients were screened and 521 were enrolled: 233 with NSCLCs, 162 with medullary thyroid carcinomas, and 76 with other tumors. The first interim analysis presented the first 114 patients with adequate follow-up (enrolled by 11 July 2019 with a data cutoff of 22 May 2020), and before that, the trial was revised. In the first version of the protocol, the investigators could enroll treatment-naïve patients only if they were judged to be ineligible for standard chemotherapy and were patients with an Eastern Cooperative Oncology Group performance status (ECOG PS) of 0-2. Among the 114 patients, 87 were previously pretreated and 27 were treatment naïve. Based on these inclusion criteria, pralsetinib showed robust activity, especially in the pretreated patients: the ORR was 61%, the mDoR was not reached, and the mPFS was 17.1 months. Importantly, the responses were irrespective of the RET fusion partner, prior multi-TKIs, and/or ICI. Among the 27 treatment-naïve patients (including those with ECOG PS 2 and up to 21% of patients tested with other tools rather than NGS and FISH), the ORR was 70%, the mDoR was 9 months, and the mPFS was 9.1 months [63,64]. The data from preclinical studies show that pralsetinib has an intracranial activity in tumor models driven by KIF5B-RET or CCDC6-RET fusions [63]. Among the 37 patients with a history of CNS metastases, the ORR was 51%, and among the 9 patients with measurable CNS disease, the intracranial ORR (IC-ORR) was 56% [62].

A second report of the trial has been published with a 17-month follow-up. In total, 281 RET + NSCLC patients were enrolled (data cutoff 6 November 2020): 75 treatment-naive patients with a median follow-up less than 12 months (47 before amendment and 28 after amendment. Of note, 28 patients enrolled after protocol revision had a lower median age, proportionally higher ECOG PS, and lower BM than those enrolled after protocol revision. There was no significant difference in toxicity among treatment-naïve and pretreated patients. In all patients, treatment discontinuation was 7%. One treatment-related death (pneumonia) occurred [64].

At the European Society for Medical Oncology (ESMO) 2022, the ARROW trial was further updated, with an additional follow-up of 16 months since the second report (data cutoff 4 March 2022). At the data cutoff, 281 RET + NSCLC (116 treatment naïve and 141 previously pretreated) (efficacy population) were enrolled, of whom 260 (107 and 130, respectively) had measurable disease (measurable disease population). Among the efficacy patient population (281), the ORR was 65.8%, the mDoR was 19.1 months, the mPFS was 13.2 months, and the OS was 44.3 months (median follow-up 25.8 months). Among the previously treated patients (141 patients), the ORR was 59.6%, the mDoR was 23.4 months, the mPFS was 16.4 months, and the OS was 44.3 months (median follow-up 28.1 months). Among the treatment-naïve patients (116 patients), 47 and 69 patients were enrolled before and after the protocol revision, respectively. Among the ‘before revision’ patients (47), the ORR was 68.1%, the mDoR was 14.7 months, and the mPFS was 10.9 months (median follow-up 29 months). Among the ‘after revision’ patients (69), the ORR was 75.4%, the mDoR was 13.4 months, and the mPFS was 13.2 months. The OS was not estimable for either group (median follow-up 19.7 months). Among the measurable diseased patient population (260), the ORR was 70%, the mDoR was 19.1 months, and the mPFS was 13.1 months (median follow-up 26.1 months). Among the previously treated patients (130 patients), the ORR was 63.1%, the mDoR was 38.8 months, and the mPFS was 14.5 months (median follow-up 29.3 months). Among the treatment-naïve patients (107 patients), 43 and 64 patients were enrolled before and after the protocol revision, respectively. Among the ‘before revision’ patients (43), the ORR was 74.4%, the mDoR was 14.7 months, and the mPFS was 11 months (median follow-up 29 months). Among the ‘after revision’ patients (64), the ORR was 79.7%, the mDoR was 12.6 months, and the mPFS was 12.6 months (median follow-up 19.7 months). Among the 15 patients with measurable CNS lesions (only 1 of which was treatment naïve), the IC-ORR was 53.3% and the mDoR was 11.5 months [65].

Among the efficacy population (281) and those with a median duration of treatment of 15 months, the AEs were 94.3%, the most common being anemia (42.3%), AST increase (44.5%), ALT increase (32.7%), neutropenia (31%), constipation (27%), hypertension (26.7%), and leukopenia (26.7%). The G ≥ 3 rate was 62.6%, and the most common G ≥ 3 AEs were anemia (19.6%), hypertension (13.9%), neutropenia (13.2%), and leukopenia 5.3%. The discontinuation rate due to AE was 10% (NSCLC) [65].

In a subgroup analysis of 233 evaluable patients for partner genes (KIF5B *n* = 164, CCDC6 n = 41, Other n = 28) plus 67 patients from the real-world data (KIF5B *n* = 46, CCDC6 n = 8, Other n = 13), responses were similar in the first two groups, but were lower in the other group (ORR around 68% vs. 39.3%). However, the long-term benefit efficacy parameters (mDoR, mPFS, and OS) were better in CCDC6/other groups versus KIF5B [66]. A subgroup analysis of pralsetinib in 68 Chinese patients (37 pretreated and 31 treatment naive) showed that the drug had similar efficacy and safety in respect to the global patient population [67].

On September 4, 2020, the Food and Drug Administration (FDA) granted accelerated approval of pralsetinib for adult patients with RET + (detected by a test approved by the FDA) NSCLCs irrespective of prior treatment lines based on these results (confirmed on August 9, 2023, as a regular approval) [68]. On December 13, 2020, the European Medicines Agency (EMA) approved pralsetinib for patients with RET + NSCLCs who had not been treated with a prior RET inhibitor [69].

Real-world data from the Italian Expanded access program (EAP) of pralsetinib confirmed the following results: among the 62 patients (13 treatment naïve) enrolled, the ORR was 66%, with an mPFS of 8.9 months. Among the six patients with CNS target lesions, the IC-ORR was 83%. AEs occurred in 83.6% of the patients and had a G > 3 incidence of 39%. The most common G3 or higher AES were neutropenia (9.8%), oral mucositis (8.2%), and thrombocytopenia (6.6%). AEs leading to dose reduction were 42%, and those leading to drug discontinuation were 12%. Two treatment-related deaths were recorded (one sepsis and one typhlitis) [70].

AcceleRET Lung (NCT04222972) was a global randomized phase III clinical trial of first-line pralsetinib versus standard chemotherapy +/− pembrolizumab (at investigator’s discretion) in 226 treatment-naïve patients with RET + advanced NSCLCs. Crossover to receive pralsetinib upon disease progression was permitted for patients randomized to the control arm. The primary endpoint was PFS, assessed using BIRC [71]. On 23 January 2024, Blueprint Medicines decided to prematurely close the clinical trial based on the decision to discontinue the global marketing and development of pralsetinib in all territories excluding the USA and China [72]. We hope that the analyses of the data collected so far for this study will be carried out and that the results can soon be published.

### 4.2. Selpercatinib

Selpercatinib (formerly known as LOXO-292) is a selective TKI of RET kinase. Preclinical studies have shown that selpercatinib inhibits several RET fusions (IC50 4 nM with selpercatinib vs. 75 nM with cabozantinib and 935 nM with vandetanib), point mutations, and acquired resistance mutations (RET V804M), avoiding interfering with other non-RET targets. In vivo, selpercatinib has been shown to inhibit the growth of RET-altered human cancer cell lines and patient-derived xenografts, including a patient-derived RET fusion + xenograft injected orthotopically into the brain. The first proofs of activity of selpercatinib towards two patients have also been described: one patient with RET-mutated (M918T) medullary thyroid carcinoma refractory to multi-TKIs (sorafenib, vandetanib, and cabozantinib) and another patient with RET fusion + NSCLC progressing on chemotherapy, erlotinib, nivolumab, and alectinib [73].

Libretto-001 (NCT03157128) was a global phase I/II trial of selpercatinib in patients with any type of solid tumor harboring an activating RET alteration. RET was tested using local testing performed in a certified laboratory with the use of NGS, FISH, or RT-PCR. During the phase I part, 57 patients were treated at seven doses (from 20 mg daily to 160 mg twice daily), observing no DLTs and establishing the recommended dose for the phase II as 160 mg twice daily [74]. The primary endpoint of the phase II part was ORR by BIRC. The first interim analysis included the first 105 previously treated patients (49 from the phase I part and 56 from the phase II part) plus 39 treatment-naïve patients (even if no power calculations were carried out in relation to the treatment-naïve patients). The baseline characteristics, except for the previous therapy, were generally similar in the previously treated and previously untreated groups, although previously untreated patients tended to have better PS and had a lower incidence of brain metastases at baseline. Among the 105 previously treated patients, the ORR was 64%, the mDoR was 17.5 months, and the mPFS was 16.5 months. Among the 39 treatment-naive patients, the ORR was 85%, and the mDoR and the mPFS were not estimable [75].

A subgroup analysis of Libretto-001 was performed among the 80 patients with CNS metastases at baseline, of whom 56% had received prior brain radiotherapy. Among the 22 patients with CNS target lesions, the IC-ORR was 82%, with the IC-mDoR not estimable. Overall, the IC-mPFS was 13.7 months [76]. Moreover, selpercatinib was demonstrated to be effective towards a female patient with EMLA4-RET + NSCLC who progressed to agerafenib (an RAF inhibitor) developing leptomeningeal carcinomatosis attaining a PR with selpercatinib with a duration of response not estimable after a follow-up of 10.8 months [77].

An update was made to the Libretto-001 trial (data cutoff 13 January 2023) including all patients enrolled before the data cutoff with at least a follow-up of 6 months. The median follow-up was around 40 months. Among the 247 pretreated patients, the ORR was 61.5%, the mDoR was 31.6 months, the mPFS was 26.2 months, and the OS was 47.6 months. Among the 69 treatment naive patients, the ORR was 82.6%, the mDoR was 20.3 months, the mPFS was 22 months, and the OS was not estimable. In total, 93 patients had CNS metastases at enrollment, and among the 26 with CNS target lesions, the IC-ORR was 84.6% and the IC-mDoR was 9.36 months [78,79].

Concerning the safety, the AEs of any grade were 95.9%, the most common AEs were dry mouth (42.8%), edema (38.4%), diarrhea (34.8%), increased AST (34.3%), increased ALT (33.7%), hypertension (28.2%), skin rash (27.3%), and fatigue 23.5%. The G ≥3 rate was 42%, and the most common G ≥3 AEs were hypertension (14.1%), ALT increase (11.6%), AST increase (6.6%), diarrhea (3%), skin rash (1.1%), edema, constipation, and fatigue (0.8% each), nausea/vomiting (0.6% each), and abdominal pain (0.3%). QTc prolongation of any grade occurred in 16.3% of the patients, of whom 4.4% G3 or higher. AEs leading to dose reductions were 48.9%, and those leading to drug discontinuation were 11% [78,79].

On 21 September 2022, the FDA approved selpercatinib for adult patients with RET fusion + NSCLCs irrespective of their prior treatment lines [80]. On 29 September 2022, the EMA approved selpercatinib for advanced NSCLCs in adults not previously treated with a RET inhibitor [81].

Table 3 summarizes the results of the phase I/II trials of pralsetinib and selpercatinib.

Libretto 431 (NCT04194944) was a randomized (2:1) phase III trial of first-line selpercatinib as compared to standard treatments (chemotherapy +/− pembrolizumab at the investigator’s discretion). The principal endpoints were PFS by BIRC in both the intention-to-treat (ITT)–pembrolizumab population and the overall ITT population. Patients were stratified according to geographic region (East Asia vs. other), brain metastases (absent vs. present), and pembrolizumab (yes vs. no). Crossover from the control group to the selpercatinib group was allowed at disease progression as assessed using BIRC. Key eligibility criteria included unresectable stage IIIB, IIIC, or IV non-squamous NSCLC, treatment naivety, ECOG PS 0-2, and RET locally tested using NGS (58% of the cases) or RT-PCR (42%), but asymptomatic or stable CNS metastases were allowed. A preplanned interim analysis was performed after 98 events of disease progression had occurred, with a data cutoff of 1 May 2023, establishing a median follow-up of 19 months. In the overall ITT- population, 261 patients were randomized (158 in the selpercatinib group and 98 in the control group), while in the ITT-pembrolizumab population, 212 patients were randomized (129 patients in the selpercatinib group and 83 patients in the control group) [82].

Baseline characteristics were well balanced in the overall ITT population, while more patients from East Asia were allocated to the selpercatinib group (58% vs. 49%) in the ITT pembrolizumab population. Overall, the most common RET fusion partners were KIF5B (45%) and CCDC6 (10%). In the ITT pembrolizumab population, the mPFS with selpercatinib was 24.8 months versus 11.2 months with standard care (HR 0.46). The benefits were consistent across all of the subgroups, including geographic region and fusion partners. The responses were higher in the experimental arm (ORR 84% vs. 65%), and mDoRs were also longer in the selpercatinib arm (24.2 vs. 11.5 months). Similar results were observed in the overall ITT population: mPFS was 24.8 months in the selpercatinib group vs. 11.2 months with the control arm (HR 0.48). Data on the overall survival rate were immature (28.6%), with 60% of patients already assigned to the control arm having crossed to selpercatinib [82].

In total, 150 out of 192 patients (from ITT pembrolizumab population) did not have CNS metastases at baseline. The 12-month cumulative incidence of CNS progression in these patients was 1.1% in the selpercatinib group vs. 14.7% with the control arm (HR 0.17), and 12-month PFS was 91.8% in the selpercatinib group vs. 74.7% with the control arm (HR 0.46). Among the 42 patients with baseline CNS metastases, 29 had brain target lesions and the IC-ORR were 81% vs. 57%, and similar trends were observed in the cumulative incidence of CNS progression and CNS-PFS [83]. These data confirm that from an integrated analysis of patients with or without CNS metastases from the LIBRETTO-001 and LIBRETTO-201 (expanded access program), cumulative incidence rates of CNS and brain progression among 30 patients with baseline CNS disease were 10%, 17%, and 20% at 12 months, 24 months, and 36, respectively, and among 31 patients without baseline, CNS disease was 0% at all cutoff points [84].

The AEs of any grade were 100% (selpercatinib) vs. 99% (control arm). The most common AEs of any grade in selpercatinib were AST increase (61%), ALT increase (60%, hypertension (48%), diarrhea (44%), edema (41%), dry mouth (39%), blood bilirubin increase (37%), skin rash (33%), and fatigue (32%). The G ≥ 3 AEs were 70% (vs. 37% in the control arm). The most G ≥ 3 AEs in the selpercatinib arm were ALT increase (22%), hypertension (20%), AST increase (13%), QTc prolongation (9%), edema, fatigue, and thrombocytopenia (3% each). AEs leading to dose reductions in the selpercatinib arm (vs control arm) were 51% (vs. 29%), and drug discontinuation was 10% (vs. 2%). Two treatment-related deaths occurred in the selpercatinib arm (sudden death and malnutrition) [82].

Table 4 depicts the results of the preplanned interim analysis of the Libretto-431 trial.

## 5. Resistance Mechanisms to TKIs

The resistance mechanisms to multi-TKIs have been well described in preclinical studies, while small clinical reports support these significant findings. On-target (those that acquired RET resistance mutations) and/or off-target (those that bypass pathway activation) have been reported despite it not being possible to determine the real impact of each alteration in determining the resistance to multi-TKIs.

### 5.1. On-Target Mechanisms

As we already mentioned above, preclinical data have already shown that gatekeeper mutations (mutations to the kinase hydrophobic pocket blocking the drugs’ access) V804 on RET could confer resistance to vandetanib [61]. A more recent preclinical study conducted on RET kinase-dependent BaF3/KIF5B-RET cells analyzed fourteen mutations in the RET tyrosine kinase domain [85]. Most of them (ten) were in the Gly-rich loop (L730, E732, and V738), the gatekeeper residue (V804) or the hinge strand (Y806, A807), and the front solvent G810 mutation, which together constitute around two-thirds of the drug binding pocket, while no mutations in the C-lobe residues have been detected. Interestingly, the researchers observed a different behavior of these mutations to four multi-TKIs (cabozantinib, vandetanib, lenvatinib, and nindetanib): Y806N conferred resistance to all the multi-TKIs studied, and the mutations associated with cabozantinib were L730I, E732K, and V871I; those associated with resistance to lenvatinib were V738A, A807V, and F998V; and the front solvent G810S was resistant to vandetanib and the L730V/804M double mutations were resistant to nindetanib. Moreover, at the same site, a different amino acid substitution could affect the behavior of this mutation to different multi-TKIs: for example, L730I caused pan resistance to the four TKIs, L730V was not associated with resistance to lenvatinib, G810S mutation caused resistance to all of the TKIs analyzed, and G810A only induced resistance to vandetanib [85]. It was only in 2018 that two different case reports reported that the acquired RET V804M gatekeeper mutation and the S904F activation loop mutation in two patients with CCDC6-RET fusion-positive NSCLCs progressed on vandetanib [86,87].

In NSCLC, with the advent of selective pralsetinib and selpercatinib, more data are being acquired on resistance to selective RET inhibitors. Both the ARROW and Libretto-001 trials showed that pralsetinib and selpercatinib efficacy was irrespective to prior multi-TKI treatment, giving an indirect proof of the clinical activity of these drugs towards the RET gatekeeper mutations potentially occurring after this prior treatment (detected only in patients with medullary thyroid carcinoma) [62,75]. A post hoc analysis from LIBRETTO-001 (selpercatinib) analyzed pre-treatment and post-progression tumor biopsies and plasma cell-free DNA (cfDNA) specimens collected from 72 patients. There was no statistically significant difference in treatment response as a function of the RET fusion partner or prior multi-TKI treatment (only four gatekeeper mutations were detected, all of which were in patients with medullary thyroid cancer). To investigate the putative innate mechanisms of resistance to pralsetinib, concomitant mutations in other genes were analyzed. Among the co-occurring mutations, only p53 mutations were associated with shorter PFS with selpercatinib treatment. Importantly, patients with baseline negative ctDNA were associated with low tumor disease burden and longer PFS with selpercatinib, while the 11 cases of PI3K alterations were not associated with sensitivity reduction to selpercatinib, and conflicting results in were found in patients with concomitant KRAS mutation (1 PD, 1 mixed response) [88]. Also, the investigators detected three patients (11%) with G810X mutations (two patients with RET fusion + NSCLC) and Y806C (one patient with medullary thyroid carcinoma)) [88].

In two patients with BIF5B-RET or CCDC6-RET + NSCLCs treated with selpercatinib, acquired heterogenous front solvent G810X (G810R, G810S, and G810C at different tumor sites) RET mutations were documented, which can sterically inhibit the binding of selpercatinib to RET fusions [89]. In a multi-center retrospective analysis of 23 post-treatment biopsies from eighteen patients with RET fusion + NSCLCs treated with selpercatinib (ten patients), pralsetinib (seven patients), or pralsetinib/selpercatinib sequential treatment (one patient) in two cases (10%) were detected the front solvent G810X RET mutations [90]. Moreover, in a patient with a CCDC6-RET fusion + NSCLC, it was detected that progressing on selpercatinib acquired a front solvent G810C/S and a new hinge region Y806C/N. An additional five non-gatekeeper RET mutations were detected in 39 cross-resistant selpercatinib/pralsetinib cell lines [91]. On the other hand, a proof of preclinical activity of selpercatinib towards pralsetinib-resistant L730V/I RET mutations has ben demonstrated [92].

### 5.2. Off-Target Mechanisms

Off-target mechanisms have been described as well. The activation of different pathways (e.g., MET and HER2 amplification) can be the cause of resistance to TKIs, as it has already been demonstrated in patients with EGFR-mutated NSCLC progressing on osimertinib [93]. Two different preclinical studies have investigated the putative role of EGF (epidermal growth factor) produced by endothelial cells in the induction of resistance to multi-TKIs [94,95]. In a small sample of patients from phase II of cabozantinib (NCT01639508), an analysis of 16 and 6 pre- and post-cabozantinib tissue biopsies detected the presence MDM2 amplification (antagonist of p53) in both pre- and post-treatment samples (around 50% each), suggesting that the amplification of MDM2 could mediate innate and acquired resistance to cabozantinib [96].

The emergence of high MET amplification occurred in a patient with KIF5B-RET fusion + NSCLC progressing on selpercatinib [97]. In the already mentioned retrospective study of Lin et al., in 18 patients treated with selpercatinib or pralsetinib, besides the front solvent G810X RET mutations (10%), MET amplification was confirmed in three cases (15%) and KRAS amplification was identified in one case (5%) [90]. The emerging occurrence of KHDRBS1–NTRK3 fusion was observed in a patient with KIF5B-RET fusion + LCNEC (large cell neuroendocrine carcinoma) progressing on selpercatinib [98]. Notably, only one case of small-cell transformation has been reported in a patient with KIF5B-RET adenocarcinoma progressing on pralsetinib [99].

In the post hoc analysis from LIBRETTO-001 (selpercatinib), the authors detected seven patients (26%) with acquired KRAS- (G12A/R/V, G13D, A59del), NRAS- (G13D, Q61R), or BRAF-activating mutations or MET or FGFR1 amplifications [88].

**Figure 2:**
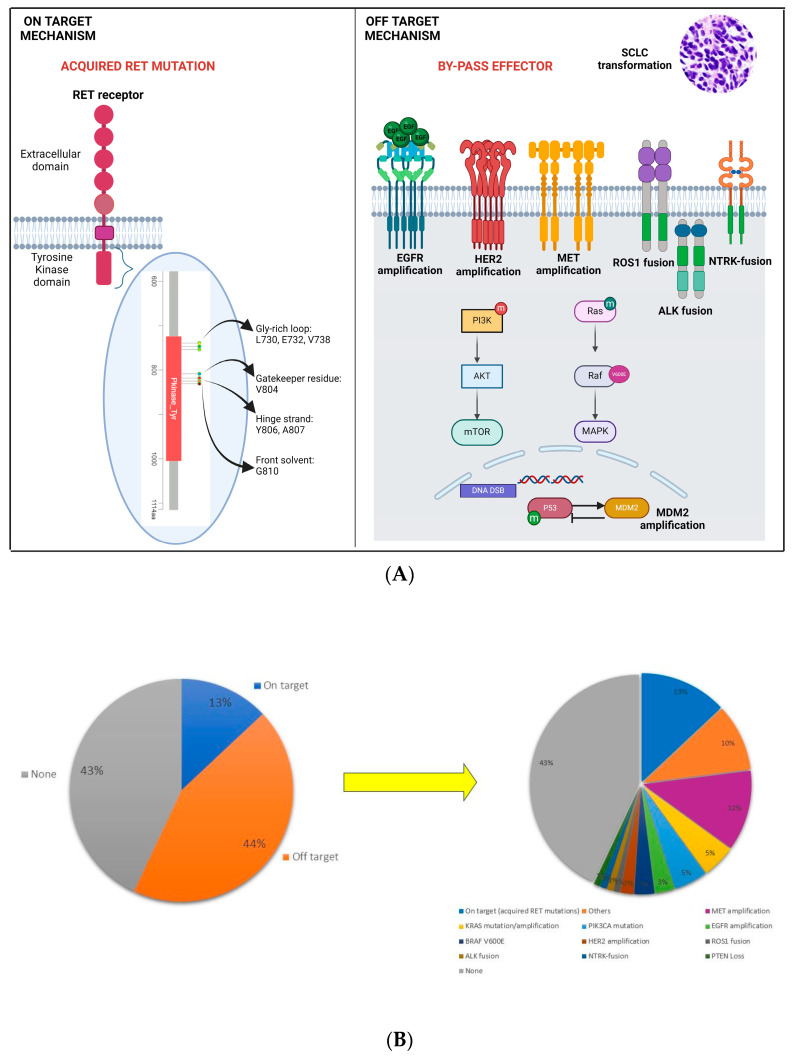
Mechanisms of resistance to RET inhibitors. (**A**) Representation of the on-target and off-target mechanisms; (**B**) pie chart depicting the different percentages of each resistance mechanism. Abbreviations: KRAS = Kirsten rat sarcoma virus gene; EGFR = Epidermal Growth Factor Receptor; HER2 = human epidermal growth factor receptor 2; ALK = Anaplastic Lymphoma kinase; PTEN = Phosphatase and TENsin homolog; MET = Mesenchymal–Epithelial Transition gene; PIK3CA = Phosphatidylinositol-4,5-Bisphosphate 3-Kinase Catalytic Subunit Alpha gene; BRAF = v-Raf murine sarcoma viral oncogene homolog B gene; ROS1 = ROS1 protooncogene; NTRK = neurotrophic tyrosine receptor kinase. Created with BioRender.com (accessed on 14 July 2024). References: [92,93].

Retrospective data from RETgistry were analyzed, with a total of 105 biopsies (61 tissue, 31 plasma, and 13 paired tissue/plasma) from 89 patients (73 with NSCLC) treated with selpercatinib (71 patients), pralsetinib (14 patients), or selpercatinib/pralsetinib sequential treatment (4 patients). At baseline, co-occurring mutations were detected in the p53 gene (29%) and CDKN2A/B (12%). On-target mechanisms (secondary RET mutations or RET gains) were detected in 13% of them, and the most common were G810X (more than 50% of all mutations, 10% of al mechanisms), V804M, A764G, V804_G810delins, G810-V804M+/-L881V in trans, or RET gains. Off-target gene alterations were detected in 46 cases (44%), and the most common were MET amplification (12%), KRAS gain or mutation (5%), activating PIK3CA mutation or PTEN loss (5%), BRAF V600E or fusion (3%), EGFR amplification (3%), ERBB2 amplification (2%), and ROS1 or ALK fusions (1% each). Importantly, no cases of small-cell transformations have been recorded in the 61 tissue samples. No differences in duration of treatment, nor PFS between on- and off-target mechanisms, were observed [100]. Figure 2A,B depicts the different mechanisms of resistance to RET-Is.

## 6. New Drugs

New selective RET inhibitors have become increasingly successful, becoming the “gold standard” as first-line therapy in patients with RET fusion + NSCLCs along with the reduction in VEGFR-related toxicities and inhibition of other kinases. However, most of the patients develop resistance to TKIs through the occurrence of acquired RET mutations in around 10–13% of cases and/or the bypass pathway activations in around 26–44% of cases [88,100]. Moreover, around 10% of patients need to discontinue the TKIs due to toxicity [65,79,82]. Clinical research is therefore aimed at designing new molecules capable of more potently and selectively inhibiting RET (including acquired RET mutations) to further improve efficacy and above all implement the therapeutic index of this class of drugs.

### 6.1. Next Generation RET TKIs

The clinical development of TPX-0046, a new RET/SRC inhibitor which shows great potency against CCDC6/RET cell lines and cell lines harboring the front solvent G810R acquired mutations, has been withdrawn due to high toxicity [101].

LOXO-260 is a highly potent and selective inhibitor of RET designed to have activity against both the solvent front and gatekeeper mutations, expressed alone or together, while maintaining potency against RET fusions or mutations [102]. LOXO-NGR-21001 is a global phase I study of LOXO-260 in patients with RET-altered solid tumors (including RET fusion + NSCLC) that must have received a prior RET inhibitor closed at the enrollment; we are awaiting the results [103].

Zeteletinib (BOS172738) is a new potent and selective RET kinase inhibitor with >300-fold selectivity against VEGFR2 to minimize the potential toxicity. In the phase I/II trial of BOS172738 (NCT03780517), the drug attained an ORR of 33% among 30 patients with RET fusion + NSCLCs with or without CNS involvement. Among the 67 patients with RET + solid tumors (safety population), the most common AEs were an increase in creatinine phosphokinase (CPK) (54%), dyspnea (34%), facial edema, AST elevation, anemia (25% each), neutropenia, diarrhea (22% each), fatigue (21%), and constipation (20%). Importantly, no ALT increase and hypertension occurred [104].

In the ongoing phase I/II trial of HA121-28 (RET/EGFR/VEGFR multi-TKI), 41 patients (part 1: 29, part 2: 12 patients) have been enrolled. Two dose-limiting toxicities (G3 anorexia, QTc prolongation) at the dose of 800 mg daily have occurred, and 600 mg daily has been chosen as the recommend dose for the phase II trial. AEs of any grade were 93%, and 6 (15%) were ≥grade 3. The most common AEs were rash (49%), diarrhea (41%), prolonged QTc interval (37%), proteinuria (20%), and nausea (20%). The ORR of RET fusion + NSCLC patients was 41%, with mDoR and mPFS not being estimable at the time of reporting [105].

SY-5007, a highly potent RET inhibitor targeting both RET fusions and mutations, in the ongoing Chinese phase I/II trial (NCT0527836) among the 105 evaluable patients showed an ORR of 77.1% (treatment naïve n = 56 ORR 84%, pretreated n = 49 ORR 69.4%), with mDoR and mPFS not being estimable after a median follow-up of 4.57 months. Among the ten patients with CNS target lesions, the IC-ORR was 80%. AEs of any grade were 96.2%, with the most common being increased AST, increased ALT, decreased neutrophil count, decreased white blood cell count, and decreased platelet count. The grade ≥ 3 AE rate was 42.9%. AEs leading to dose interruption were 39%, those leading to dose reduction were 23.8%, and those leading to drug discontinuation were 1.9% [106].

In an ongoing Chinese phase I/II trial of HS-10365 (NCT05207787), 31 RET fusion + NSCLC patients with RET TKI-naivety were treated with HS-10365 at six doses (40 mg QD to 200 mg BID). The MTD was not defined, and 160 mg of BID was chosen as the recommended phase II dose. The most common AEs were an increase in AST, bilirubin, and ALT, a decrease in WBC, PLT, and neutrophil, an increase in serum creatinine, QTc prolongation, hypoalbuminemia, and anemia. No treatment discontinuations have been reported. The ORR was 70% (66.7% among the 24 pretreated patients and 83.3% among the 6 treatment-naïve patients [107].

EP0031 (A400/KL590586) is highly selective for RET compared to other kinases and has showed preclinically greater potency than first-generation TKIs towards common RET aberrations including acquired RET resistance mutations. Moreover, it was shown that EP0031 could reach higher brain exposure compared to first TKIs in intracranial xenograft models. Two phase I/II trials of EP0031 are ongoing in China (NCT05265091) and the western countries (NCT05443126). The preliminary results of the Chinese phase I/II trial analyzed the first 109 patients with RET + solid tumors treated with EP0031 at al doses (10–120 mg daily), reporting no DLTs [108]. AEs of any grade were 94.5%, and the most common AEs were an increase in AST (51.4%) and ALT (48.6%), constipation (31.2%), creatinine increase (30.3%), and headache (30.3%). The Grade ≥ 3 rate was 23.9%, consisting of the following: anemia 2.8%, increase in AST (1.8%), ALT (1.8%), headache 0–9%, creatinine (0.9%), and bilirubin (0.9%). AEs of special interest were hypertension 4.6% (Grade ≥ 3 0%), hyponatremia 7.3% (0.9%), and QTc prolongation 2.8%. AEs leading to dose interruptions were 36.7%, those leading to dose reductions were 6.4%, and those leading to drug discontinuation were 2.8%. Among the 59 patients with RET fusion + NSCLC, the ORR was 80.8% and 69.7% in the 26 treatment-naïve patients and the 33 previously pretreated patients, respectively. The mDoR was not estimable. Five out of seven patients previously treated with first TKIs and treated with EP0031 at 90–120 mg doses had a partial response. Among the six patients with CNS target lesions, the IC-ORR was 83.3% (5/6) [108]. At the ASCO 2024 meeting, the updated results of the Western Phase I/II trial were presented (NCT05443126) [109,110]. In total, 35 patients were evaluable for safety, and 24 (12 with NSCLC) patients for efficacy. No MTD was reached, and 90mg QD was selected as the RP2D, with plasma levels > IC90 for all of the relevant RET fusions/mutations, including resistance mutations. The most common AEs were headache, constipation, dizziness, ALT, AST, blurred vision, anemia, dry mouth, nausea, and back pain. Grade 3 treatment-related or not-treatment-related AEs reported were the following: hyponatremia (four patients), headache (two patients), hypertension (two patients), anemia (two patients), diarrhea (two patients), AST (one patient). The ORR was 50% (6/12) in the 12 patients with RET fusion + NSCLCs. Four out of ten patients previously pretreated with first-generation TKIs had a PR, and two out of five patients with CNS metastases attained a global ORR. Phase II is ongoing regarding NSCLC, medullary thyroid carcinoma, or other solid tumor patients with or without prior TKI treatment [110].

TY-1091, a second-generation RET inhibitor, shows sub-nanomolar potency inhibition of RET fusions and activity towards acquired RET single or double mutations [111]. A Chinese phase I/II trial of TY-1091 (NCT05675605) is ongoing, with no results available at present. Vepafestinib (TAS0953/HM06) is another new RET inhibitor that shows a high potency towards RET fusions and acquired mutations and a high brain-barrier penetrance in animal models [112]. APS03118 selectively targets RET fusions and mutations, including solvent front mutations, G810C/S/R, and the gatekeeper V804 mutation. A Chinese phase I trial of APS03118 is ongoing (NCT05675605).

A preclinical study conducted in human medullary thyroid carcinoma cell lines demonstrated that adefovir dipivoxil, a drug already approved for the treatment of chronic hepatitis B, inhibited RET gene transcription, reducing the expression of endogenous RET proteins [113]. At the present, there are no current clinical trials investigating adevofir in patients with RET fusion + NSCLC, but this drug could be useful to overcome acquired RET resistances and mitigate the RET-I-related toxicities.

Table 5 depicts the ongoing clinical trials of new RET-Is.

### 6.2. New Strategies

A retrospective multicenter study of 225 patients with RET fusion + NSCLCs investigated the role of bevacizumab adding to CT +/− ICI. Among the 185 patients treated with platinum-based CT, there was a statistically significant longer mPFS and OS in the group treated with bevacizumab (20 patients) compared to patients treated with CT (97 patients) or CT + ICI (68 patients): mPFS (17 months vs. 8.8 months, *p* = 0.018; 17 months vs. 9.7 months, *p* = 0.05); OS (51.4 vs. 38.7 months). Unusually, among the 40 patients treated with mono-CT, the addition of bevacizumab (8 patients) seemed to be detrimental: ORR 13% vs. 25%, mPFS 2.4 months vs. 4.1 months, OS 12.1 vs. 39 months [114].

As already reported, MET or EGFR amplifications can mediate resistance to TKIs up to 12% and 3%, respectively, of the cases [100]. A phase I/II trial (NCT05845671) of amivantamab (a bifunctional antibody anti-EGFR/MET) plus TKI is ongoing for 35 patients with NSCLC harboring ALK, ROS1, and RET fusions progressing on FDA-approved TKIs for their respective oncogene. Participants must be on their TKI at the same dose for at least 3 months prior to enrolling in the study, in combination with a standard fixed dose of common ALK, ROS1, and RET TKIs. The primary endpoint will be the objective response rate (ORR) [115].

Lung MAP (NCT05364645 US phase II trial) is investigating the role of continuing a RET inhibitor (selpercatinib) beyond disease progression by adding standard chemotherapy to the TKI.

A randomized phase III trial of sacituzumab–tirumotecan (an antibody-drug conjugated anti-TROP2 pay-linked with a topoisomerase inhibitor) compared to CT (docetaxel or pemetrexed) in 556 patients with NSCLC harboring EGFR mutation or other molecular drivers (including RET alterations) after progressing on first-line TKI (NCT06074588) is ongoing.

Two Chinese retrospective studies are investigating the putative role of ICI administered alone in second-line or further settings after progressing on TKI in 186 patients with gene-addicted NSCLC (NCT04777175) and in combination with CT (compared to CT alone) as first-line settings in 70 patients with RET fusion NSCLCs (POSEIDON NCT04322591).

Table S3 summarizes all of the ongoing clinical studies in patients with RET fusion + advanced/metastatic NSCLCs (including other novel drugs such as bispecific antibodies, combinations strategies, and new developing ICIs) [116].

### 6.3. Early Stage

LIBRETTO-432 (NCT04819100) is an ongoing phase III, global, randomized (1:1), double-blind trial to test selpercatinib vs. placebo for 3 years in the adjuvant setting in 170 patients with RET fusion + Stage IB-IIIA NSCLC following the surgery. The primary endpoint is investigator-assessed event-free survival (EFS) in the primary analysis population (patients with stage II-IIIA), and, if positive, will be evaluated using EFS assessed by investigators in the overall population (patients with stage IB-IIIA) and OS in both the primary analyses and overall populations [117]. BO42777 (NCT05170204) is an ongoing phase I–III platform study evaluating the safety and efficacy of multiple targeted therapies versus durvalumab following CT-RT in patients with unresectable stage III oncogene-addicted (ALK, ROS1, RET) NSCLCs. Key inclusion criteria include locally advanced unresectable stage III NSCLC, ≥2 prior cycles of concurrent or sequential CT-RT, and ECOG PS 0–2. Patients are stratified based on staging, CT-RT type (concomitant or sequential), and PD-L1 status. The primary endpoint is PFS as assessed using blinded central review [118].

Table S4 summarizes the ongoing clinical trials in patients with RET fusion + NSCLCs in the early stage (including a neoadjuvant trial, the adjuvant trial LIBRETTO-432, and maintenance therapy after a curative CRT in the III stage).

## 7. Toxicity

The selective RET-Is, selpercatinib and pralsetinib, in addition to improving clinical efficacy, should reduce the toxicity by not interfering with other kinases, especially VEGFR. While we are aware of the inadequacy of cross-trial comparisons, which above all in this regard concern different sample sizes (small phase II/retrospective studies for multi-TKIs) and have notable differences in the median duration of treatment between multi-TKIs and selective inhibitors, we attempted to analyze the most important safety endpoints to confirm the safety gain obtained with selective inhibitors. The G ≥ 3 toxicity rate with multi-TKI treatment ranged from 71% to 92% (we have excluded two trials of vandetanib and ponatinib due to a too-small sample and short follow-up) [49,53], while those dosed with pralsetinib were 62.6% and those with selpercatinib were 42% (LIBRETTO-001) and 70% (LIBRETTO-431) [45,48,51,65,79,82]. Considering the worst case scenario, the reduction in G3 or higher toxicity with selective RET-Is was approximately 15% (mean multi-TKIs 82% vs. RET-Is 66.3%). The AEs leading to dose reduction ranged from 28% to 73% (mean 55.8%) with multi-TKIs vs. 38% (pralsetinib) to 51% (selpercatinib, LIBRETTO-431) (mean 44.5%), allowing us to reduce the needs for dose reduction by around 11% [45,48,49,51,65,79,82]. Even more importantly, AEs leading to drug discontinuation ranged between 8% and 24% (two studies of vandetanib and ponatinib did not report the data) (mean 17.6%) with multi-TKIs vs. 10–11% with RET-Is (mean 10.5%), with a gain on average of around 7.1% [45,48,49,51,65,79,82].

If we analyze the most common toxicities of RET-Is, the caveats for selpercatinib are liver enzymes and hypertension, and those for pralsetinib are hypertension and neutropenia.

TKI-induced liver toxicity can be related to several factors such as metabolic factors (mitochondrial disfunction and hepatocyte apoptosis induction) and/or immune-mediated factors, and its incidence varies greatly among the different TKIs [119,120]. Importantly, liver toxicity can be distinguished as intrinsic (dose dependent) and idiosyncratic (dose independent) [121]. Regarding the TKIs, all of them can cause an increase in liver enzymes, but only cabozantinib (G ≥ 3 = 8%), and above all selpercatinib (G ≥ 3 = 11.6%), are associated with severe toxicity [45,79,82]. In the LIBRETTO-001, liver toxicity was among the main causes of adverse events leading to drug discontinuation [79]. Moreover, the first-line trial of selpercatinib (LIBRETTO-431) recorded an even higher rate of G≥ 3 AST/ALT increase of 13%/22% [82].

Hypertension is one of the most common AEs of VEGFR inhibitors. This toxicity seems to be associated with vasoconstriction due to the decreased production of nitric oxide [122]. Hypertension is a well-known toxicity of bevacizumab (32%, G ≥ 3 = 17%) and first-generation multi-TKIs sunitinib (28%, G ≥ 3 = 6%) and sorafenib (17%, G ≥ 3 = 3%) [123,124,125]. Concerning the multi-TKIs used in the RET fusion NSCLC, hypertension is most prominent in vandetanib and lenvatinib compared to cabozantinib [45,48,49,51]. Both selective RET-Is caused hypertension with a G ≥ 3 of around 15–20% [65,79]. The possible explanations could be that both drugs inhibit VEGFR at the clinical doses administered or that RET inhibition itself can mediate hypertension.

Another typical side-effect of VEGFR-inhibition is proteinuria caused by VEGF inhibition on podocytes and the loss of endothelial fenestrations in glomerular capillaries [126]. The incidence of proteinuria in bevacizumab treatment is around 23% [123]. Besides the trial of lenvatinib [51], which reported an incidence of 48% (G ≥ 3 = 16%), the other studies did not observe this AE, but it may have been strongly underestimated since it is an asymptomatic event that must be detected with a urine test, which was not mandatory in all of the studies. Therefore, we strongly recommend monitoring proteinuria through urine analysis during selpercatinib/pralsetinib treatment.

Another potential underestimated AE could be represented by impaired wound healing (around 13% with bevacizumab treatment) that will have to be taken into account, especially in clinical studies of RET-Is for perioperative treatment [127]. As we have already mentioned, RET is expressed by hematopoietic stem cells, and the neurotrophic ligands are produced in in the hematopoietic stem cell environment [20]. Based on this physiological condition, the potent RET inhibition by pralsetinib and selpercatinib can cause medullary toxicity, which is significantly more pronounced than multi-TKIs. In detail, in the ARROW trial, neutropenia leading to treatment interruption was reported in 39 (17%) patients and was reported as leading to dose reduction in 28 (12%) patients. Only one (<1%) patient discontinued treatment, owing to neutropenia. Four (2%) patients had febrile neutropenia, but none of these events resulted in treatment discontinuation [62].

An important warning for drugs that potentially inhibit the VEGF pathway is the gastrointestinal perforation that, even as a rare event (less than 2%), can be life-threatening and can be explained by the regression in capillaries of intestinal villi in mouse models [127,128]. At present, no cases of gastrointestinal perforation have been observed. Only one patient treated with cabozantinib died due to a retroperitoneal hemorrhage [45]. In the phase II trial of lenvatinib, patients suffered from abdominal pain (3%), and especially in the two trials of selpercatinib, abdominal pain has been experienced in 11.1% (G ≥ 3 = 0.4%) (LIBRETTO-001) and in 25% (G ≥ 3 = 1%) (LIBRETTO-431) of patients [51,79,82]. Two case reports showed that one patient with RET fusion positive NSCLC and ten patients with RET-mutated medullary thyroid carcinoma treated with selpercatinib experienced intestinal edema [129,130].

Even more severe AEs are represented by chylous effusion or ascites (CE or CA). Eight cases have been described in patients with medullary thyroid carcinoma during selpercatinib treatment [131]. A retrospective analysis of a pan-cancer cohort of 7517 patients treated with multi-TKIs plus 96 patients treated with RET-Is (pralsetinib, selpercatinib) showed that 15 patients developed CE (12 patients chylothorax, 5 patients CA, 5 both). CE were the most common in the selpercatinib group (7%), followed by agerafenib (4%), cabozantinib (0.3%), and lenvatinib (0.02%); none were observed with pralsetinib [132]. Two further cases were reported in a patient with a KIF5B-RET fusion + NSCLC treated with selpercatinib and in another patient with a CCDC6-RET fusion + NSCLC treated with pralsetinib [133]. CA is a peritoneal effusion characterized by a cream- or milk-like fluid consistency with a triglyceride level over 200 mg/dL [134]. The pathogenesis is not well known. One plausible explanation involves the microscopic disruption of lymphatic channels, rather than the direct obstruction of the thoracic duct, as the etiological factor behind chylous effusion [132]. An accurate examination of patients who suffer from dyspnea or thoracic/abdominal pain is mandatory in patients treated with these drugs, and in cases including the diagnosis of an intestinal edema or CE, must be indicated with pharmacovigilance. Management of CA consists of a temporarily interrupted RET-TKI dose which is then started over at a reduced dose, a high-protein low-fat diet, and monthly octreotide injections; for severe cases, invasive measures (retreated paracentesis) are suggested [133].

AEs of special interest are QTc prolongation for selpercatinib and pneumonitis for pralsetinib. QTc prolongation seems to be peculiar to vandetanib (47.4% G ≥ 3 = 10.5%) and selpercatinib (16.3% G ≥ 3 = 4.4%). None of these events developed fatal arrythmia [48,75,79]. Pneumonitis (in some cases described as pneumonia) has been observed during lenvatinib treatment (four cases, 16% all G≥ 3) and above all pralsetinib [51,65]. The incidence of pneumonitis was 12%; of these cases, 2% were grade 3 or higher. Serious AEs were reported in 55 (24%) patients, and pneumonitis (10 patients (4%)) or pneumonia (10 patients (4%)) were among the main causes. No cases resulted in fatal death [65].

**Figure 3:**
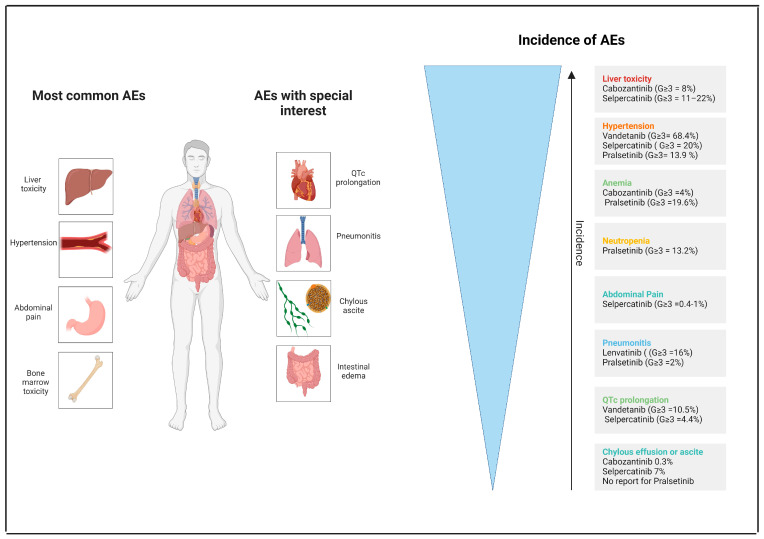
Representation of the most common adverse events (AEs) and AEs with special interest. Created with BioRender.com (accessed on 14 July 2024. Abbreviations: AEs = adverse events; G3 = grade 3; QT = QT interval.

Table 6 and Figure 3 summarizes the toxicities related to these TKIs.

## 8. Discussion

RET gene fusions were discovered in NSCLCs just over 10 years ago [22]. Their incidence in NSCLCs is relatively rare, being around 1–2% of all NSCLCs [26]. Key clinical features of patients with RET fusion + NSCLCs are like other fusion-driven diseases: younger age, adenocarcinoma histology, low exposure to tobacco, and with a tendency to spread to the brain (one quarter of the cases with advanced disease at the diagnosis) [28,29,30].

Retrospective data have shown that CTs and ICIs have a low impact on the clinical outcomes of patients with this disease [32,33,34,35,38,39,41,42,43]. The multi-TKIs, already approved for other diseases, have achieved only modest activity in RET fusion + NSCLCs, with an ORR ranging from 16 to 53%, but their clinical use has been severely limited by their high toxicities and is very likely related to VEGFR2 and other targets of inhibition [45,47,48,49,51,53].

The advent of more potent and selective RET-Is (pralsetinib and selpercatinib) dramatically changed the clinical scenario, achieving higher responses (ORR 70–80%), a mPFS of around 2 years, and showing high efficacy in the management of CNS metastases in patients regardless of the presence or absence of CNS metastases at baseline [65,75,79]. Both the FDA and the EMA approved pralsetinib and selpercatinib in first-line settings already based on the two phase I/II trials ARROW and LIBRETTO-001, respectively [68,69,80,81]. Moreover, two global randomized trials are currently investigating the role of selpercatinib and pralsetinib in early-stage and locally advanced settings, respectively [117,118].

So, striking results have been achieved in very few years. So why did we decide to title the review ‘shadows and fogs’ (a title inspired by Woody Allen’s black-and-white film of the same name)? Because there are not only lights, there are lights and shadows. If we only consider the safety profile of the RET-Is, we could go so far as to say ‘shadows and fogs’. In fact, if it is true that the main safety endpoints (G ≥3 AE, dose reduction, and drug discontinuation rates) have improved with the use of RET-Is compared to multi-TKis, there are some toxicities correlated to the inhibition of VEGFR-2 (hypertension primarily), and there others whose mechanisms are not well known (also attributable to the RET inhibition itself), with non-negligible rates of grade 3 or higher, such as the following: hypertension (both drugs), medullary toxicity (pralsetinib, thrombocytopenia, both drugs), pneumonitis (pralsetinib), and QTc prolongation (selpercatinib). One factor worthy of particular mention is the abdominal toxicities (intestinal edema and chylous ascites), which are extremely rare, but are potentially very severe and have been described in patients with thyroid cancer and also, in some cases, lung cancer [130,132].

In our opinion, since these tumors are relatively rare and these events are extremely sporadic, a close pharmacovigilance can help better quantify these events, define their severity, and help us to understand the possible pathogenetic mechanisms [135]. Moreover, an integrated analysis of safety data of RET-Is among patients with RET fusion + NSCLC with those with medullary thyroid carcinoma and other tumors should be mandatory to define if some toxicities are only drug-dependent or can be linked also to a specific disease [136,137].

On the other hand, like all targeted drugs, patients treated with RET-Is also develop disease progression after a relatively long beneficial period. The resistance to RET-Is can be mediated by the acquired RET mutations (on-target) (around 10–13%) and bypass pathway activation (around 26–44%) mainly due to MET amplification or KRAS mutation or amplification [88,100]. Therefore, clinical research is focusing on designing new molecules capable of more potently and selectively inhibiting RET to overcome RET secondary mutations and also in defining new combinations of drugs to target the different pathways involved in the resistance to RET-Is. At the present, among the next generation of RET inhibitors (Loxo-260, EP0031, TY-1091, and vepafestinib) capable of overcoming the RET-acquired mutations in the preclinical models, only EP0031 has given proof of activity in patients pretreated with RET-Is in two distinct phase I trials (5/7 patients in the Chinese phase I trial, and 4/10 patients in the Western phase I/II trial) [103,106,108,110,116]. Moreover, other new drugs (selective RET inhibitors or multi-targeted inhibitors) (zeteletinib, HA121-28, SY-5007, and HS-10365) have shown clinical safety and activity in treatment-naïve and pretreated patients with RET fusion + NSCLCs, but not in patients previously pretreated with RET-Is [104,105,106,107]. On the other hand, two distinct ongoing trials are exploring the safety and activity of a bifunctional antibody (amivantamab) anti-EGFR/MET in association with RET-I in patients progressing on RET-Is (NCT05845671) or a new ADC anti-TROP2 (sacituzumab-tirumotecan) in patients with oncogene-addicted NSCLCs (including RET fusions) (NCT06074588).

## 9. Conclusions

The advent of new RET-Is (pralsetinib, selpercatinib) have improved the prognosis and treatment landscape of patients with RET fusion + NSCLCs. Clinical research must be focused on developing new drugs with activity towards the acquired RET mutations and with higher selectivity, sparing other targets (VEGFR2 and others) and new combination strategies to further improve the clinical results together with a sustained improvement in the safety profile.

## Figures and Tables

**Table 1:** Retrospective studies of ICIs in patients with RET fusion + NSCLCs.

Study (Reference)	Patient Number	Line	ORR (%)	mPFS (Months)	OS (Months)
Offin 2019 [38]	16	≥1 L	0	3.4	NR
Yan 2024 [39]	38	1 L + 2 L *	26.3	5.0	19.0
Immunotarget (Mazieres 2019) [41]	16	≥2 L	6	2.1	21.3
Dudnik 2018 [42]	13	≥1 L	0	3.0	14.9
GFPC 01-2018 (Guisier 2020) [43]	9	≥2 L	37.5	7.6	NE

Legend: * 17 patients in first-line and 21 patients in second-line settings. Abbreviations: ORR = overall response rate; mPFS = median progression-free survival; OS = median overall survival; L = line; NR = not reported; NE = not evaluable.

**Table 3:** Phase I/II trials of selective RET inhibitors (pralsetinib, selpercatinib) in patients with RET fusion + NSCLCs.

Drug	Trial	Setting	Pt N	ORR		mPFS (Months)		OS (Months)	IC ORR *	G ≥ 3	Reduction Rate	Discontinuation Rate
**Pralsetinib**	**Ph I/II ARROW** (281 pts)	≥2 L	141	59.6%		16.4		44.3	53.3%	62.6%	38%	10%
		1 L	116	72.4%		12.6		NE	-			
		B 47	A 69	68%	75%	10.9	13.2	NE I NE	-			
**Selpercatinib**	**Ph I/II Libretto001** (316 pts)	≥2 L	247	61.5%		26.2		47.6	84.6%	42%	48.9%	11%

Legend: * IC ORR in 15 patients (ARROW trial) and 26 patients (Libretto-001 trial) with CNS target lesions. Abbreviations: Pt N = patient number; ORR = overall response rate; mPFS = median progression-free survival; OS = median overall survival; IC ORR = intracranial ORR; G = grade; Ph = phase; pts = patients; B = before protocol revision; A = after protocol revision.

**Table 4:** Results of the preplanned interim analysis in the intention to treat pembrolizumab population of Libretto-431 trial (selpercatinib vs. chemotherapy +/− pembrolizumab).

Trial	Arm	Pt N	ORR	mDoR (Months)	mPFS (Months)	IC-ORR	12-mo CNS inc *	G ≥ 3	Reduction Rate	Discontinuation Rate
**Libretto-431** Zhou 2023 [82]	**Selpercatinib**	129	84%	24.2	24.8	82%	6%	70%	51%	10%
					HR 0.46					
	**CT + pembro**	83	65%	11.5	11.2	58%	20%	57%	29%	2%

Legend: * please note that here we have reported the 12-month CNS cumulative incidence in all patients regardless of CNS metastases at the baseline. Noteworthy: In the overall intention-to-treat population (261 patients), 129 patients were randomized to selpercatinib and 98 to CT +/− pembro: the mPFS was 24.8 months with selpercatinib vs. 11.2 months with control arm (HR 0.48). Abbreviations: Pt N = patient number; ORR = overall response rate; mDoR = median duration of response; mPFS = median progression-free survival; IC-ORR = intracranial ORR; 12-mo CNS inc = 12-month cumulative CNS incidence; G = Grade; HR = Hazard Ratio.

**Table 5:** Ongoing clinical trials of new RET-TKIs.

Drug	Target(s)	Phase	Tumor	N. Patient	ORR	Trial Identifier	Comment
TPX-0046	RET (G810R+) SRC	I/II	RET + advanced solid tumors	41	-	NCT04161391	Drug withdrawal for toxicity.
Loxo-260	RET (G810X+)	I	Unresectable locally advanced or metastatic cancers RET +	110	-	NCT05241834	Active, not recruiting.
	RET (G810X+)	EAP	RET + advanced solid tumors	NA	-	NCT05225259	Expanded access program no longer available.
BOS172738 Zeteletinib Schöffski 2021 [104]	RET	I	RET + advanced solid tumors	67	33%	NCT03780517	Dose: 150 mg QD. Trial completed. Last update 30 October 2023. No further trials planned.
HA121-28 Zhao 2021 [105]	RET/EGFR/VEGFR	I/II	RET + advanced solid tumors	41	41%	NCT03994484	Chinese study. Included 11 patients with NSCLC in phase II.
HA121-28	RET/EGFR/VEGFR	II	RET + aNSCLC (No prior RET-Is)	83 *	-	NCT05117658	Primary endpoint: ORR. Location: China. Unknown status enrollment.
SY-5007 Xiong 2024 [106]	RET	II	RET + aNSCLC	All pts (105 pts) Treatment naïve (56 pts) Pretreated pts (49)	77.1% 83.9% 69.4%	NCT05278364	In phase I, included all metastatic solid tumors.
SY-5007	RET	III	RET + aNSCLC (Treatment naïve, RET fusion on tissue or lx)	120 *	-	NCT06031558	Chinese non-randomized trial in first-line setting. Active, recruiting.
HS-10365 Lu 2023 [107]	RET	I/II	RET + advanced solid tumors	All pts (30) # Pretreated pts (24) Treatment naïve pts (6)	70% 66.7% 83.3%	NCT05207787	An amount of 160 mg BID was the RP2D. Active, recruiting.
EP0031 (A400/KL590586) Zhou 2023 [108]	RET	I	RET + advanced solid tumors	All pts (57) # Treatment naïve (25 pts) Pretreated (32pts)	80.8% 69.7% 50%	NCT05265091	Chinese population. Active, not recruiting.
EP0031 Garralda 2024 [110]	RET	I/II	RET + advanced solid tumors	12 pts with NSCL (10 pts preteated)	50%	NCT05443126	Western population. Active and recruiting.
TY-1091	RET	I/I	RET + advanced solid tumors	248 *	-	NCT05675605	Primary endpoint: DLT/ORR. Location: China. Active and recruiting.
TAS0953/HM06 (Vepafestinib)	RET	I/II	RET + advanced solid tumors (prior RET-Is allowed)	202 *	-	NCT04683250 (MARGARET)	Primary endpoint: RP2D/ORR. Location: Japan–USA. Active and recruiting.
HEC169096	RET	I/II	RET + advanced solid tumors (no restriction on prior treatment lines)	456 *	-	NCT05451602	Primary endpoint: RP2D/ORR. Location: China. Active and recruiting.
HS-10365	RET	I/II	RET + aNSCLC (no prior RETIs)	62 *	-	NCT06147570	Primary endpoint: RP2D/ORR. Location: China. Active and recruiting.
APS03118	RET	I	RET + advanced solid tumors	35 *	-	NCT05653869	Location: China. Active and recruiting.

Legend: * patients estimated in the planned enrollment; # we reported the results of advanced NSCLCs. Abbreviations: ORR = overall response rate; mPFS = median progression-free survival; RET = rearranged during transfection; SRC = proto-oncogene tyrosine-protein kinase; aNCSLC = advanced or metastatic non-small cell lung cancer; PR = partial response; CR = complete response. Treatment = treatment; lx = liquid biopsy; DLT = dose-limiting toxicity; NA = not available.

**Table 6:** Safety profile of multi-tyrosine kinase inhibitors and RET inhibitors.

Parameter	Multi-TKIs	RET-Is
**G ≥ 3 AE rate (mean)**	82%	66.3%
Gain	−15.7%	
**Dose reduction (mean)**	55.8%	44.5%
Gain	−11.3%	
**Drug discontinuation (mean)**	17.6%	10.5%
Gain	−7.1%	
**Specific AEs**		
**G3 ≥ liver toxicity**	8% (cabozantinib)	13–22% (selpercatinib)
**G ≥ 3 hypertension**	68.4% (vandetanib)	13.9–20% (pralsetinib/selpercatinib)
**G ≥ 3 Anemia**	4% (cabozantinib)	19.6% (pralsetinib)
**G ≥ 3 Neutropenia**	-	13.2% (pralsetinib)
**G ≥ 3 Thrombocytopenia**	4%/8% (lenvatinib/cabozantinib)	3%/4% (selpercatinib/pralsetinib)
**Pneumonitis (G ≥ 3)**	16% (16%) (lenvatinib)	12% (2%) (pralsetinib)
**QTc prolongation (G ≥ 3)**	47.4% (10.5%) (vandetanib)	16.3% (4.4%) (selpercatinib)
**Chylous ascites (G: NA)**	-	7% (selpercatinib)

Abbreviations: Multi-TKIs = multi-tyrosine kinase inhibitors; RET-Is = RET (rearranged during transfection) inhibitors; G = grade; AE(s) = adverse event(s); NA = not applicable.

## Supplementary Materials

The following supporting information can be downloaded at: https://www.mdpi.com/article/10.3390/cancers16162877/s1, Table S1. Chemotherapy in RET + NSCLC. Table S2. Clinical trials and retrospective studies of multi-tyrosine kinase inhibitors in patients with RET fusion + NSCLC. Table S3. ongoing clinical trials in patients with RET altered NSCLC. Table S4. Ongoing clinical trials of RET-Is in early stage and locally advanced RET-fusion positive NSCLC patients.
