# Supplementary material for: Non-Small-Cell Lung Cancers (NSCLCs) Harboring RET Gene Fusion, from Their Discovery to the Advent of New Selective Potent RET Inhibitors: “Shadows and Fogs”
Source: Cancers (Basel). 2024 Aug 19;16(16):2877. doi: 10.3390/cancers16162877 (PMC11352804; doi:10.3390/cancers16162877)
Supplement: Supplementary file 1 [file cancers-16-02877-s001.zip › cancers-3134659-supplementary.pdf]

Non-Small-Cell Lung Cancer (NSCLC) harboring RET gene fusion, from its discovery to the advent of new selective potent RET inhibitors: 'shadows and fogs'

Gianluca Spitaleri [1]*, Pamela Trillo Aliaga [1], Ilaria Attili [1], Ester Del Signore [1], Carla Corvaja [1], Gloria Pellizzari [2,3], Jalissa Katrini [2,3], Antonio Passaro [1] and Filippo de Marinis [1]

Supplementary tables

Table S1. Chemotherapy in RET+ NSCLC.

Study	Pt N (total)	Regimen	ORR	mPFS	OS
Retrospective Drilon 2016	18 (104)	Pemetrexed alone or in combination	45%	19 months	Not reached
GLORY registry Gautschi 2017	65 (84)	Platinum-based CT	51%	7.8 months	24.8 months
Retrospective Takeda 2019	1	Pemetrexed	PR	30 months	NA
Retrospective Shen 2020	40	First-line: pemetrexed vs non-pemetrexed	50% vs 44.4%	9.2 months vs 5.2 months	26.4 months*
Retrospective Shen 2020	28	Second-line: pemetrexed vs non-pemetrexed	NR Vs NR	4.9 months Vs 2.8 months	-

*MS was available for 38 patients**. Abbreviations**: Pt N = patient number; CT= chemotherapy; ORR = overall response rate; mPFS = median progression-free survival; OS = median overall survival; NR = not reported.

Table S2. Clinical trials and retrospective studies of multi-tyrosine kinase inhibitors in patients with RET fusion + NSCLC.

Drug	Reference	Phase	Pt N	ORR	mPFS	OS
Cabozantinib	Nokihara 2019	I	2	50%	NE	NE
	Gautschi 2017	R	19	27%	3.6 months	4.9 months
Vandetanib	Gautschi 2017	R	11	18%	2.9 months	10.2 months
	Platt 2015	R	3	0%	NE	NE
Lenvatinib	Gautschi 2017	R	2	50%	NE	NE
Ponatinib	Gautschi 2017	R	2	0%	NE	NE
Agerafenib (RXDX-105)	Drilon 2019	I	31	19%	NE	NE
Sorafenib	Horiike 2016	II	3	0%	NE	NE
	Gautschi 2017	R	2	0%	NE	NE
Alectinib	Lin 2016	R	4	25%	NE	NE
	Gautschi 2017	R	2	0%	NE	NE
	Ribeiro 2020	R	4	0%	NE	NE
Sunitinib	Gautschi 2017	R	9	22%	2.2	6.8
Regorafenib	Gautschi 2017	R	1	0%	NE	NE
Nindetanib	Gautschi 2017	R	2	50%	NE	NE

Abbreviations: Pt N = patient number; ORR = overall response rate; mPFS = median progression-free survival; OS = overall survival; R = retrospective study; NE = not estimable.

Table S3. Ongoing clinical trials in patients with RET altered NSCLC.

Trial Identifier	Phase	Pt N	Drug(s)	Setting	Principal endpoint	Region	Study completion estimated

NCT01639508	II	86	cabozantinib	RET+, ROS1+ or NTRK+ NSCLC	ORR	USA	Jul 2026 (No further publication since 2016)
LIBRETTO-001 NCT03157128	I/II	875	Selpercatinib	RET+ solid tumors	RP2D/ORR	Worldwide	28 Feb 2026
**Combo**							
NCT05845671	I/II	12	Amivantamab + TKI	ALK+, ROS1+, RET+ NSCLC (after TKI)	RP2D/ORR	USA	Jan 2028
LungMAP NCT05364645	II	74	Selpercatinib + CT	RET+ NSCLC (progressing on RET-I as last tx)	PFS	USA	1 May 2029
NCT06074588	III	556	Sacituzumab Tirumotecan vs CT (docetaxel or pemetrexed)	EGFR or other driver NSCLC (second line)	PFS	America, Australia, Israel, East Asia	11 Mar 20230
**ICI**							
NCT04777175	R	186	ICI	Gene-addicted NSCLC	PFS	China	12 Jan 2025
POSEIDON NCT04322591	Obs	70	CT vs CT+ ICI	RET+ NSCLC (1st-line)	PFS	China	24 Mar 2025

Abbreviations: ICI = immune checkpoint inhibitors; CT = chemotherapy; NSCLC = Non-Small-Cell Lung Carcinoma; pCR = pathological complete response; CRT = chemo/radiotherapy; DFS = disease-free survival; PFS = progression-free survival; RET = rearranged during transfection; ROS1 = ROS proto-oncogene 1, receptor tyrosine kinase; NTRK = Neurotrophic tyrosine receptor kinase; ORR = overall response rate; RP2D = recommended phase II dose; RET-I(s) = RET inhibitor(s); DLT = dose-limiting toxicity; Tx = treatment; lx = liquid biopsy; ALK = anaplastic lymphoma kinase; TKI = tyrosine kinase inhibitor; EGFR = Epidermal Growth Factor Receptor. **Reference**: https://clinicaltrials.gov/search?cond=NSCLC&term=RET&aggFilters=status:not%20rec&viewType=Table. Last accessed on 27 Jun 2024.

Table S4. Ongoing clinical trials of RET-Is in early stage and locally advanced RET-fusion positive NSCLC pts.

Trial Identifier	Phase	Pt N	Drug(s)	Setting	Principal endpoint	Region	Study completion estimated
NCT05800340	II	30	Neo-adjiuvant ICI (toripalimab) + CT	Rare gene-addidcted cIIB-IIIA NSCLC	pCR	China	31 Dec 2026
LIBRETTO-432 NCT04819100	III	170	Selpercatinib after surgery for 3yr vs Placebo (+/- adjuvant CRT)	RET fusion NSCLC stage IB-IIIA	EFS (II-IIIA)	Worldwide	30 Aug 2032
BO42777 NCT05170204	III	320	Pralsetinib for 3yr Vs Durvalumab for 1yr	Unresectable stage III, gene addicted (ALK+, ROS1+, RET+) NSCLC	BIRC PFS	Worldwide	14 Apr 2035

Legend: *Note for ALK+ alectinib, ROS1 + entrectinib. **Abbreviations:** RET = rearranged during transfection; NSCLC = Non-Small-Cell Lung Carcinoma; ALK = Anaplastic lymphoma kinase; ROS1

= ROS proto-oncogene 1; EFS = investigator assessed event-free survival; BIRC PFS = blinded independent central review progression-free survival.
